# Risk of Non-Hodgkin Lymphoma among Patients with Hepatitis B Virus and Hepatitis C Virus in Taiwan: A Nationwide Cohort Study

**DOI:** 10.3390/cancers14030583

**Published:** 2022-01-24

**Authors:** Yung-Rung Lai, Ya-Lan Chang, Chiu-Hsiang Lee, Tung-Han Tsai, Kuang-Hua Huang, Chien-Ying Lee

**Affiliations:** 1 Department of Pharmacy, Chung Shan Medical University Hospital, Taichung 40201, Taiwan; cshd050@gmail.com (Y.-R.L.); cshd141@csh.org.tw (Y.-L.C.); 2 School of Nursing, Chung Shan Medical University, Taichung 40201, Taiwan; csha528@csh.org.tw; 3 Department of Nursing, Chung Shan Medical University Hospital, Taichung 40201, Taiwan; 4 Department of Health Services Administration, China Medical University, Taichung 40402, Taiwan; dondon0525@gmail.com (T.-H.T.); khhuang@mail.cmu.edu.tw (K.-H.H.); 5 Department of Pharmacology, Chung Shan Medical University, Taichung 40201, Taiwan

**Keywords:** hepatitis B virus, hepatitis C virus, non-Hodgkin lymphoma

## Abstract

**Simple Summary:**

Non-Hodgkin lymphoma (NHL) is difficult to diagnose and has a high mortality rate. Large-scale database research is necessary to examine and strengthen the correlation between viral hepatitis and NHL. This retrospective cohort study analyzed differences in the risk of developing NHL for patients with hepatitis to elucidate these relationships by using nationwide data from Taiwan’s National Health Insurance Research Database. In this study, the incidence rate of NHL in patients with hepatitis B was 0.22%, and in patients with hepatitis C, the incidence rate of NHL was 0.35%. These comparisons indicate that patients with HBV or HCV have a higher incidence of NHL (OR, 2.37; 95% CI, 1.93–2.91).

**Abstract:**

Hepatitis B virus (HBV) and hepatitis C virus (HCV) are associated with an increased risk of developing non-Hodgkin lymphoma (NHL); however, adequate data corroborating these associations are lacking. Therefore, a study based on the national database was performed to investigate the correlation between HBV and HCV with NHL in Taiwan. This research was a retrospective cohort study using a nationally representative database established by the Health and Welfare Data Science Center of the Ministry of Health and Welfare, Taiwan. The participants were patients with HBV and HCV, analyzed using the propensity score matching method. The study results indicated that the incidence rate of NHL (0.13%) was significantly higher than that in patients from the general population. After controlling related variables, the hazard ratio (HR) of the incidence of NHL in patients with hepatitis was 2.37 (95% CI, 1.93–2.91). Furthermore, the incidence of NHL in patients with HBV was significantly higher than in patients from the general population (HR, 2.49; 95% CI, 1.94–3.19). The incidence of NHL in patients with HCV was significantly higher than in patients from the general population (HR, 2.36; 95% CI, 1.73–3.22). This study indicated that HBV and HCV significantly increase the risk of NHL.

## 1. Introduction

Hepatitis B virus (HBV) and hepatitis C virus (HCV) infections are key public health problems worldwide. It has been reported that HBV and HCV are relatively common in Taiwan [1,2]. Taiwan has been controlling viral hepatitis B infections through a large-scale vaccination campaign. The anti-HBV surface antigen (HBsAg) carrier rate in children in Taipei has dropped from 11% to 0.5% between 1984 and 2014 [3]; another surveillance study indicated that the estimated age-adjusted HCV seroprevalence rate was about 3.28% in Taiwan [2].

Non-Hodgkin lymphoma (NHL) is the most common malignant lymphoid tumor, and incidence rates of NHL vary widely by world region [4]. The cause of NHL is currently unclear. Studies have indicated that the cause of NHL may be related to viral or bacterial infection or poor immune function [5,6]. Additionally, many studies have shown that chronic liver disease can also increase the risk of developing NHL [7,8,9,10,11]. Preventing NHL among patients with HBV and HCV requires special attention.

In southern and eastern Europe, Japan, and the southern United States, NHL and HCV have been reported to be highly correlated. In contrast, in central and northern Europe, Canada, the northern United States, and some countries in Asia, no correlation has been reported. The relatively weak odds ratios of NHL for HCV infection may explain the notable inconsistencies in the literature. The inconsistent results of these studies are likely because of differences in study design, geography, and the ethnicities of the study participants [8,12,13].

NHL is difficult to diagnose and has a high mortality rate. Large-scale database research is necessary to examine and strengthen the correlation between viral hepatitis and NHL. This retrospective cohort study analyzed differences in the risk of developing NHL for patients with HBV and HCV to elucidate these relationships by using nationwide data from Taiwan’s National Health Insurance Research Database.

## 2. Materials and Methods

### 2.1. Database

In this study, secondary data were analyzed, examining information from 2000 to 2016 obtained from the Longitudinal Health Insurance Database (LHID), which was established by Taiwan’s Ministry of Health and Welfare (MOHW). The LHID contains data on the medical records and causes of death for 2 million randomly sampled participants in Taiwan’s National Health Insurance (NHI) program. Taiwan’s NHI program has enrolled up to 99% of Taiwanese residents since 1995. Hence, the LHID represents the utilization of healthcare in Taiwan. The LHID contains anonymous data to protect the privacy of beneficiaries and is maintained by the Health and Welfare Data Science Center. Therefore, the requirement for informed consent was waived for this study, and the study protocol was approved after ethical review by the Institutional Review Board of China Medical University Hospital, Taiwan (No: CMUH107-REC2-004).

### 2.2. Study Participants

In this study, patients newly diagnosed as having HBV or HCV between 2002 and 2013 were selected as the research group, to ensure that each participant had undergone a follow-up period of at least 3 years [14]. Hepatitis was defined as the main diagnosis in 3 or more outpatient clinic visits within one year for HBV (International Classification of Diseases, Ninth Revision [Tenth Revision], Clinical Modification [ICD-9-CM]: 070.20–070.23, 070.30–070.33; ICD-10-CM: B16, B17.0, B18.0, B18.1, B19.1) or HCV (ICD-9-CM: 070.41, 070.44, 070.51, 070.54, 070.7, V02.62; ICD-10-CM: B17.10, B17.11, B18.2, B19.2, Z22.52) and the concurrent use of chronic hepatitis medications, including interferon and L-nucleoside agents.

We selected patients from the general population as a control group for comparison. To reduce outcome bias and avoid selection bias, we used the propensity score matching (PSM) method, which entailed patient and control matching by age and sex at a ratio of 1 (patients with chronic hepatitis) to 5 (patients from the general population) to obtain comparisons for increasing the comparability between the hepatitis cohort and the control population [15,16]. To increase the accuracy of the results, we excluded those patients who had been diagnosed with NHL before chronic hepatitis. We assigned the comparison the same index date as the hepatitis cohort, according to a matching identification. Furthermore, patients who received NHL diagnoses before the index date were also excluded to reduce research bias.

**Figure 1:**
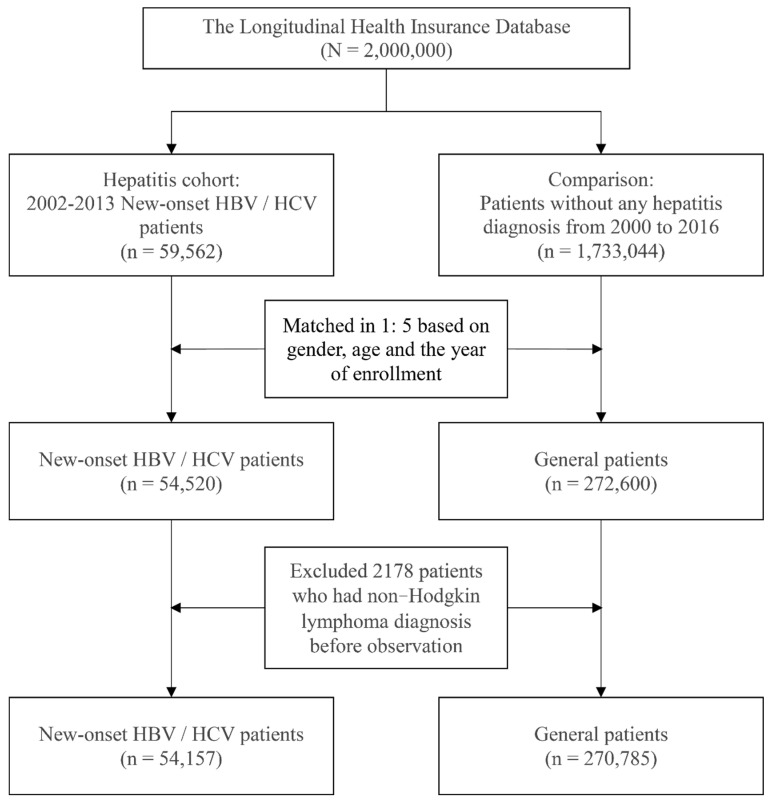
Screening process for the study subjects.

A total of 324,942 participants were included from 2002 to 2013, of whom 54,157 were patients with HBV or HCV, and 270,785 were patients from the general population. The screening process for the selection of research participants is shown in Figure 1.

### 2.3. Study Design

The study design was a retrospective cohort study to examine the risk of NHL in patients with HBV or HCV. The date of diagnosis of chronic hepatitis was defined as the observation start date for participants in the study group, and after matching, the same date was assigned as the observation start date for corresponding members of the control group. All participants were tracked from the observation start date until death, or the participant being diagnosed with NHL, or the end date of the study. The definition of NHL in the study was based on ICD-9-CM diagnostic code 202.8 and ICD-10-CM diagnostic codes C85.8 and C85.9. The control variables in this study were the patient’s sex, age, and related comorbid conditions. The comorbid conditions included diabetes mellitus (ICD-9-CM: 250), hypertension (ICD-9-CM: 401–405), dyslipidemia (ICD-9-CM: 272), kidney disease (ICD-9-CM: 580–588), rheumatoid arthritis (RA) (ICD-9-CM: 714), lupus erythematosus (LSE))(ICD-9-CM: 710), psoriasis (ICD-9-CM: 696), human immunodeficiency virus (HIV) (ICD-9-CM: 042–044), and organ transplant (NHI surgery order codes: 68035, 68037, 68047, 75020, 76020, 75418, 85213).

### 2.4. Statistical Analysis

All analyses in the study were performed using SAS version 9.4, and statistical significance was defined as *p*-values < 0.05. Descriptive statistics were used to analyze the numbers and percentages of patient characteristics (sex, age, and health status) and other variables; chi-squared analysis was performed to compare the differences in various variables. The Cox proportional hazards model was used to determine the relationship between the occurrence of NHL for patients with HBV or HCV by controlling the relevant variables.

## 3. Results

### 3.1. The Baseline Characteristic Distribution of Study Subjects after Matching

This study examined the occurrence of NHL in patients newly diagnosed with hepatitis between 2002 and 2013. After excluding patients who had previously been diagnosed with NHL, a total of 324,942 participants were included, 54,157 of whom were patients with hepatitis and 270,785 were patients from the general population. Table 1 lists the distribution of the variables of the study participants. Patients were matched by sex and age, and chi-squared tests were performed to determine any differences between the patients with hepatitis and paired patients from the general population. No statistically significant difference (*p* > 0.05) was observed in the matching variables, including gender and age.

### 3.2. The Incidence Rate of Non-Hodgkin Lymphoma

Table 2 presents the bivariate analysis for each variable and the associated occurrence of NHL. Among all patients with hepatitis, the HBV group comprised 37,656 patients (69.53%), the HCV group comprised 14,365 patients (26.52%), and 2136 patients were diagnosed as having both HBV and HCV. A total of 419 patients from this study developed NHL, and the overall incidence rate of NHL was 0.13%. The incidence rate of NHL in patients with hepatitis was 0.25%, which was significantly higher than the incidence rate of NHL in patients from the general population, which was 0.10%. For the variable “age”, older patients exhibited a higher incidence of NHL. The incidence rate of NHL in patients ≥ 65 years was 0.28%, which was significantly higher than that in patients aged 20 to 44 (0.07%) and 45 to 54 (0.07%). For patients with comorbidities, the highest incidence of NHL was 1.35%, which was for patients who had received allografts. For patients with diabetes and hypertension, the respective incidence rates of NHL were 0.17% and 0.16%; for patients with rheumatoid arthritis, the incidence rate of NHL was 0.32%; the incidence rate of NHL in patients with lupus erythematosus was 0.33%; the incidence rate of NHL in patients with psoriasis was 0.33%; and the incidence rate of NHL in patients with human immunodeficiency virus was 0.66%. All of the above differences were statistically significant (*p* < 0.001).

### 3.3. Risk of Non-Hodgkin Lymphoma in Hepatitis Patients

Table 3 displays the risk analysis of NHL in patients with hepatitis. After controlling other related variables, the relative risk (hazard ratio [HR]) of patients with hepatitis developing NHL was 2.37 times higher (95% CI, 1.93–2.91). The relative risk of developing NHL increased with increasing age. For example, compared with patients aged 20 to 44, the relative risk of developing NHL for patients 55 to 64 years old was 3.30 times higher (95% CI, 2.29–4.75), and the relative risk for developing NHL in patients ≥ 65 years old was 6.28 times higher (95% CI, 4.26–9.26). In the comorbidity analysis, patients with lupus erythematosus, human immunodeficiency virus, and organ transplant exhibited higher relative risks of developing NHL (HR, 1.82; 95% CI, 1.17–2.83; HR, 7.09; 95% CI, 2.62–19.22; HR, 6.59; 95% CI, 2.92–14.88, respectively). Patients with rheumatoid arthritis and psoriasis had a higher relative risk of developing NHL, but no statistically significant difference was observed (HR, 1.58; 95% CI, 0.97–2.57; HR, 1.43; 95% CI, 0.71–2.88); patients with dyslipidemia and kidney disease had lower risk of developing NHL (HR, 0.69; 95% CI, 0.54–0.87; HR, 0.64; 95% CI, 0.46–0.90); patients with diabetes and hypertension also had a lower risk of developing NHL, but no statistically significant difference was observed (HR, 0.98; 95% CI, 0.77–1.26; HR, 0.94; 95% CI, 0.75–1.26). Analysis of the patients with hepatitis entailed further division of the patients into groups of those with HBV and HCV (model 2 in Table 3). After controlling other related variables, compared with general patients, the relative risk of in patients with HBV developing NHL was 2.49 times higher (95% CI, 1.94–3.19), and for patients with HCV, the relative risk was 2.36 times higher (95% CI, 1.73–3.22). For patients with HBV and HCV, a higher relative risk of NHL was observed, but this was not statistically significant (HR, 1.20; 95% CI, 0.45–3.23).

## 4. Discussion

In this study, the incidence rate of NHL in patients with HBV was 0.22%, and in patients with HCV, the incidence rate of NHL was 0.35%. These comparisons indicate that patients with HBV or HCV have a higher incidence of NHL (OR, 2.37; 95% CI, 1.93–2.91). This study was a retrospective cohort follow-up study that used secondary database analysis to screen patients with HBV and HCV through disease diagnosis codes and prescribed medications, and used the PSM method to obtain a control group. In the investigation of the two hepatitis conditions and the occurrence of NHL, we determined that patients with HBV and patients with HCV both had a higher risk of developing NHL and that the higher relative risk between the groups occurred in patients with HBV.

The mechanism of HBV infection in NHL is still unclear. One hypothesis is that HBV can directly infect lymphocytes and be incorporated into the host genome, leading to the overexpression of oncogenes or downregulation of tumor suppressor genes. Additionally, viral hepatitis B replication and viral antigens may also induce the expression and release of hematopoietic tumor growth factors, leading to the proliferation of cloned lymphocytes [17]. HCV may indirectly affect B-cells and regulate oncogenic transformation through intracellular viral proteins. Such indirect effects include chronic antigen stimulation by HCV and the existence of potential antigen-selection-driven processes in the development of NHL in patients with HCV [18].

Further evidence of the indirect effect of HCV on NHL is the upregulation of the tumor necrosis factor family during chronic HCV infection [19]. The HCV genome produces structural (nucleocapsid, E1, E2) and nonstructural proteins that may result in the E2 protein of HCV causing chronic antigen-driven polyclonal B-cell proliferation [20]. Although the existence of HCV lymphocytes is disputed, CD81 on B-cells is a branded HCV internalization receptor, and the costimulatory receptor B7.2 (CD86) mediates HCV to memory B-cells [21]. HCV produces nitric oxide synthase and reactive-oxygen-induced DNA damage [22] and upregulates host B-cell receptor signaling in patients with HCV [23]. However, the underlying carcinogenic mechanisms are still unclear.

Taborelli et al. (2016) conducted a case–control study in Italy and reported that 3.7% of patients with NHL also developed HBV, which was a rate significantly higher than the 1.7% observed in the control group [24]. However, studies have produced inconsistent results, which may be due to differences in research designs, ethnicities, geography, and environments [12,13,25]. To increase the accuracy of the results in the present study, the selected participants with hepatitis had all been prescribed antiviral medications for chronic hepatitis; this confirmed the positive correlation between HBV infection and the development of NHL in the Taiwanese population.

A meta-analysis comprehensively assessed the association between HBV and NHL. The pooled estimates of 58 studies included the risk of NHL in patients with HBV, which significantly increased the overall odds ratio (sOR, 2.50; 95% CI, 2.20–2.83) regardless of study design (case–control study: sOR, 2.47; 95% CI, 2.16–2.82; cohort study: sOR, 2.64; 95% CI, 1.78–3.91) [26]. Previous studies examining the link between HBV infection and NHL have yielded conflicting results. Studies from Europe, Japan, China, and South Korea have indicated HR ranges from 1.74 to 4.87 [11,27,28]. However, other studies have found no significant association between HBV infection and NHL [13,29,30]. Studies in Taiwan have also corroborated the association between hepatitis infection and NHL [31,32]. Kleinstern et al. published a case–control study in 2016 to examine the relationship between HBV and the occurrence of NHL. The study included healthy participants as the control group and reported that the relative risk of developing NHL in patients with HBV was 2.39 times higher (95% CI, 1.13–5.06). Although patients with HBsAg antibodies were also determined to have a low relative risk of developing NHL (OR, 0.68; 95% CI, 0.51–0.92), the risk was not statistically significant for natural antibody responses (OR, 0.76; 95% CI, 0.56–1.04) [33]. This showed a link between persistent HBV infection and B-NHL. Another study reported that acquired immunity from natural infections also increases the risk of B-NHL. Therefore, regardless of whether HBsAg is cleared, the risk of B-NHL is likely to be higher in patients with hepatitis [34].

Studies and meta-analyses have identified an association between HCV infection and the development of B-NHL. The estimated risk of lymphoma development is moderate, with an average odds ratio between two and three. However, this estimate varies widely and depends not only on the type of histology considered, but also on the geographic locations and ethnicities of the populations included in the trials [35].

A nested large-population case–control study in Korea investigated the suspected relationship between hepatitis and NHL. The results reported that the incidence of HBV and HCV in the NHL group (3.3% and 1.3%, respectively) was higher than that in the control group (0.9% and 0.3%, respectively; *p* < 0.001) [11]. In a previous study in Taiwan, chronic HCV infection was temporally associated with a twofold increase in the risk of lymphoid tumors from 2001 to 2005, especially the risk of NHL. Although 8.2% of participants received interferon-based treatment, the statistical results of the study were not significant due to the small number of participants [14].

The relative risk of developing NHL increases with increasing age. For example, compared with patients aged 20 to 44, the relative risk of developing NHL for patients aged 55 to 64 years old was 3.30 times higher (95% CI, 2.29–4.75), and the relative risk for developing NHL in patients ≥ 65 years old was 6.28 times higher (95% CI, 4.26–9.26). Among older adults, a registry-based case–control study for HCV infection and the risk of cancer indicated a positive association with diffuse large B-cell lymphoma (aOR, 1.57; 95% CI, 1.34–1.84) [36]. One study reported the adjusted ORs of HBV in patients with NHL who were <55 years compared with those who were ≥55 years—2.28 (*p* = 0.038) and 3.48 (*p* < 0.001), respectively—and the adjusted ORs for HCV in patients with NHL who were <55 years compared with those who were ≥55 years were 2.58 (*p* = 0.114) and 3.24 (*p* = 0.044), respectively [11]. The results of the present study indicate that patients with lupus erythematosus, human immunodeficiency virus, and organ transplant were all at a higher risk of developing NHL. This confirms the correlation between immunodeficiency and NHL. The cause of NHL may be related to viral infection, bacterial infection, or weakened immune function [6]. Early research also demonstrated that several risk factors were associated with lymphoma, including immunosuppression, several autoimmune disorders (rheumatoid arthritis, celiac disease, systemic lupus erythematosus, and Sjögren’s syndrome), and certain infectious agents for NHL [37].

It has been indicated that serum HBV DNA and HCV RNA amounts reveal complex virological profiles which may be present in HBV and HCV co-infected patients [38]. In our study, patients co-infected with both HBV and HCV had a lower HR compared with HBV- and HCV-infected patients that may be due to having too few participants with NHL (n = 4). However, 95% CI is not statistically significant. The lower risk of NHL in the co-infected patients may be different depending on the geographical region and the study population. Large-scale prospective studies are warranted to assess the association between patients co-infected with both HBV and HCV and the subsequent development of NHL.

The International Agency for Research on Cancer assessed and confirmed the carcinogenicity of seven viral agents to humans, including HCV. HBV and HCV are indirect carcinogens, producing their effects through chronic inflammation [39]. A previous case–control study and meta-analysis demonstrated that chronic HBV infection was positively associated with NHL. However, acquired immunity (HBsAg−, anti-HBs+, anti-HBc+) by natural infection also increased the risk of developing B-NHL (adjusted OR, 2.25; 95% CI, 1.96–2.57) [34]. Vaccination against HBV and HCV may reduce the risk of hepatitis-related liver disease and NHL [11]. In addition, recent studies have indicated that anti-HBV and anti-HCV drugs for the treatment may contribute to the treatment of NHL [40,41,42].

Future experimental studies to further clarify the causal relationships between NHL and HBV and HCV infection may support the clinical necessity of such therapies. Although anti-HBV and anti-HCV agents have an impact on cancer risk, this study used HBV and HCV disease diagnostic codes in conjunction with medication usage to determine to a strict correlation between viral hepatitis medications and the risk of cancer in the participants. However, the main purpose of this study was to examine the risk of cancer in patients with HBV and HCV. Therefore, no further analysis of medications was necessary. We recommend that future studies further analyze and study the effects of various viral hepatitis medications on NHL.

The results of this study confirmed that patients with HBV or HCV have a higher risk of developing NHL, which is consistent with the results of previous studies. Compared with related studies, the present study has several strengths. First, we used a national sampling database for analysis. The MOHW database comprises a stratified random sample of two million individuals with information on sex, age, and location. The database is nationally representative and also avoids bias in the selection of research participants. Previous investigations concerning the hepatitis virus and NHL have mostly been case–control studies. This study, however, adopted the design of a generational follow-up study, which is a comparably more rigorous design. Finally, in addition to controlling risk factors such as sex, age, and the related comorbidities of the participants, subjects who had previously been diagnosed as having NHL were excluded before study participants were selected. PSM for the study and control groups further reduced sample selectivity bias and confirmed the representativeness and reliability of the results of this study.

This study also has limitations. The study was limited to the information provided in the LHID; information regarding patient living environments, tobacco and alcohol use, dietary habits, stress levels, and family disease history, laboratory parameters (e.g., HBV DNA, HCV RNA, and genotypes), among others, is unavailable in the database but may affect NHL factors. The LHID was also missing data regarding the active chronic hepatitis, cirrhosis, and NHL histology (such as B- or T-origin, indolent or aggressive, and the World Health Organization [WHO] classification subtypes). This study reduced confounding via adjusting comorbidity disease, but clinical situations (e.g., RA, SLE, and HIV infection) could be associated with lymphoma development. Future studies are warranted to evaluate the effects of hepatitis virus infections on the occurrence of NHL, or NHL histology after excluding related autoimmune diseases. Additionally, future research should be supplemented by clinical empirical medicine and by linking other databases or questionnaires to more rigorously and thoroughly analyze correlations or causality. Hepatitis prevention and treatment is a public health issue that Taiwan has prioritized, and cancer prevention has always been a priority medical issue in Taiwan. The results of this study may provide relevant units and clinical medical personnel guidance on hepatitis prevention and treatment strategies and serve as a reference for the future development of cancer prevention and treatment strategies for patients with hepatitis.

## 5. Conclusions

HBV and HCV infections play a significant role in the development of NHL. In patients with HBV and patients with HCV, both receiving antiviral agents, chronic coinfection with HBV and HCV was associated with an increased risk of NHL in a Taiwanese population. In addition, the relative risk of developing NHL increases with increasing age and some comorbidities (such as HPL, CKD, SLE, HIV, and organ transplant).

## Figures and Tables

**Table 1:** The baseline characteristics of patients with hepatitis after matching in a 1:5 ratio.

Variables	Control Group		Case Group		Total		*p*-Value ^3^
	Patients without Hepatitis		Patients with Hepatitis				
	n	%	n	%	n	%	
Total	270,785	100	54,157	100	324,942	100	
Gender ^1^							1.000
Female	114,960	42.45	22,992	42.45	137,952	42.45	
Male	155,825	57.55	31,165	57.55	186,990	57.55	
Age (years) ^1^	48.59 ± 15.39		48.78 ± 14.28		48.62 ± 15.21		1.000
20–44	49,060	18.12	9812	18.12	58,872	18.12	
45–54	94,555	34.92	18,911	34.92	113,466	34.92	
55–64	85,985	31.75	17,197	31.75	103,182	31.75	
≥65	41,185	15.21	8237	15.21	49,422	15.21	
DM ^2^							<0.001
No	223,161	82.41	40,342	74.49	263,503	81.09	
Yes	47,624	17.59	13,815	25.51	61,439	18.91	
HTN ^2^							<0.001
No	178,351	65.86	32,619	60.23	210,970	64.93	
Yes	92,434	34.14	21,538	39.77	113,972	35.07	
HPL ^2^							<0.001
No	199,223	73.57	35,406	65.38	234,629	72.21	
Yes	71,562	26.43	18,751	34.62	90,313	27.79	
CKD ^2^							<0.001
No	251,014	92.70	47,747	88.16	298,761	91.94	
Yes	19,771	7.30	6410	11.84	26,181	8.06	
RA ^2^							<0.001
No	266,645	98.47	52,658	97.23	319,303	98.26	
Yes	4140	1.53	1499	2.77	5639	1.74	
SLE ^2^							
No	265,912	98.20	52,396	96.75	318,308	97.96	
Yes	4873	1.8	1761	3.25	6634	2.04	
Psoriasis							<0.001
No	267,992	98.97	53,389	98.58	321,381	98.90	
Yes	2793	1.03	768	1.42	3561	1.10	
HIV ^2^							<0.001
No	270,562	99.92	53,774	99.29	324,336	99.81	
Yes	223	0.08	383	0.71	606	0.19	
Organ transplant							<0.001
No	270,594	99.93	53,903	99.53	324,497	99.86	
Yes	191	0.07	254	0.47	445	0.14	

^1^ Variables for propensity score matching. ^2^ Abbreviations: DM, diabetes mellitus; HTN, hypertension; HPL, hyperlipidemia; CKD, chronic kidney disease; RA, rheumatoid arthritis; SLE, systemic lupus erythematosus; HIV, human immunodeficiency virus. ^3^ Used chi-squared test to examine the distribution of the characteristics.

**Table 2:** Covariates associated with non-Hodgkin lymphoma with univariate analysis.

Variables	Non-Hodgkin Lymphoma				Total		*p*-Value ^2^
	No		Yes				
	n	%	n	%	n	%	
Total	324,523	99.87	419	0.13	324,942	100	
Hepatitis							<0.001
No	270,503	99.90	282	0.10	270,785	83.33	
Yes	54,020	99.75	137	0.25	54,157	16.67	
Hepatitis types							<0.001
Hepatitis B	37,573	99.78	83	0.22	37,656	11.59	
Hepatitis C	14,315	99.65	50	0.35	14,365	4.42	
Both	2132	99.81	4	0.19	2136	0.66	
Gender							0.422
Female	137,766	99.87	186	0.13	137,952	42.45	
Male	186,757	99.88	233	0.12	186,990	57.55	
Age (year)	48.59 ± 15.39		48.77 ± 14.28		48.62 ± 15.21		<0.001
20–44	58,833	99.93	39	0.07	58,872	18.12	
45–54	113,386	99.93	80	0.07	113,466	34.92	
55–64	103,021	99.84	161	0.16	103,182	31.75	
≥65	49,283	99.72	139	0.28	49,422	15.21	
DM ^1^							0.003
No	263,187	99.88	316	0.12	263,503	81.09	
Yes	61,336	99.83	103	0.17	61,439	18.91	
HTN ^1^							<0.001
No	210,749	99.9	221	0.1	210,970	64.93	
Yes	113,774	99.83	198	0.17	113,972	35.07	
HPL ^1^							0.960
No	234,326	99.87	303	0.13	234,629	72.21	
Yes	90,197	99.87	116	0.13	90,313	27.79	
CKD ^1^							0.193
No	298,383	99.87	378	0.13	298,761	91.94	
Yes	26,140	99.84	41	0.16	26,181	8.06	
RA ^1^							<0.001
No	318,902	99.87	401	0.13	319,303	98.26	
Yes	5621	99.68	18	0.32	5639	1.74	
SLE ^1^							<0.001
No	317,911	99.88	397	0.12	318,308	97.96	
Yes	6612	99.67	22	0.33	6634	2.04	
Psoriasis							0.110
No	320,970	99.87	411	0.13	321,381	98.90	
Yes	3553	99.78	8	0.22	3561	1.10	
HIV ^1^							<0.001
No	323,921	99.87	415	0.13	324,336	99.81	
Yes	602	99.34	4	0.66	606	0.19	
Organ transplant							<0.001
No	324,084	99.87	413	0.13	324,497	99.86	
Yes	439	98.65	6	1.35	445	0.14	

^1^ Abbreviations: DM, diabetes mellitus; HTN, hypertension; HPL, hyperlipidemia; CKD, chronic kidney disease; RA, rheumatoid arthritis; SLE, systemic lupus erythematosus; HIV, human immunodeficiency virus. ^2^ Used chi-squared test to exam the characteristics distribution.

**Table 3:** Risk of non-Hodgkin lymphoma in hepatitis patients with multivariable analysis of Cox regression analysis.

Variables	Model 1			Model 2		
	HR ^1^	95% CI	*p*-Value	HR ^1^	95% CI	*p*-Value
Hepatitis						
No (ref.)	1			1		
Yes	2.37	1.93–2.92	<0.001	-		
Hepatitis B	-		-	2.49	1.94–3.19	<0.001
Hepatitis C	-		-	2.36	1.73–3.22	<0.001
Both	-		-	1.20	0.45–3.23	0.720
Gender						
Female (ref.)	1			1		
Male	1.06	0.87–1.29	0.567	1.06	0.87–1.29	0.574
Age (year)						
20–44 (ref.)	1			1		
45–54	1.29	0.87–1.89	0.203	1.29	0.88–1.90	0.194
55–64	3.30	2.29–4.75	<0.001	3.33	2.31–4.81	<0.001
≥65	6.28	4.26–9.26	<0.001	6.37	4.31–9.42	<0.001
DM ^1^						
No	1			1		
Yes	0.98	0.77–1.26	0.893	0.99	0.77–1.27	0.928
HTN ^1^						
No	1			1		
Yes	0.94	0.75–1.18	0.584	0.94	0.75–1.18	0.586
HPL ^1^						
No	1			1		
Yes	0.69	0.54–0.87	0.002	0.68	0.54–0.86	0.002
CKD ^1^						
No	1			1		
Yes	0.64	0.46–0.90	0.010	0.64	0.46–0.90	0.011
RA ^1^						
No	1			1		
Yes	1.58	0.97–2.57	0.066	1.59	0.98–2.58	0.063
SLE ^1^						
No	1			1		
Yes	1.82	1.17–2.83	0.008	1.82	1.17–2.83	0.008
Psoriasis						
No	1			1		
Yes	1.43	0.71–2.88	0.317	1.43	0.71–2.89	0.316
HIV ^1^						
No	1			1		
Yes	7.09	2.62–19.22	<0.001	7.39	2.72–20.10	<0.001
Organ transplant						
No	1			1		
Yes	6.59	2.92–14.88	<0.001	6.67	2.96–15.05	<0.001

^1^ Abbreviations: DM, diabetes mellitus; HTN, hypertension; HPL, hyperlipidemia; CKD, chronic kidney disease; RA, rheumatoid arthritis; SLE, systemic lupus erythematosus; HIV, human immunodeficiency virus.

## Data Availability

The National Health Insurance Database used to support the findings of this study was provided by the Health and Welfare Data Science Center, Ministry of Health and Welfare (HWDC, MOHW) under license and so cannot be made freely available. Requests for access to these data should be made to HWDC (https://dep.mohw.gov.tw/dos/cp-5119-59201-113.html, accessed on 11 November 2021).
